# Coping Strategies Used by Newly Appointed Nurse Educators During Transition from Clinical Practice to Academia: A Qualitative Study

**DOI:** 10.3390/nursrep15100367

**Published:** 2025-10-15

**Authors:** Tumelo Dintwe, Gopolang Gause, Leepile Alfred Sehularo

**Affiliations:** 1 NuMIQ Research Focus Area, Faculty of Health Sciences, North-West University, Potchefstroom 2520, South Africa; tumelo.dintwe@nwu.ac.za; 2 Lifestyle Diseases Research Focus Area, Faculty of Health Sciences, North-West University, Mafikeng 2745, South Africa; leepile.sehularo@nwu.ac.za

**Keywords:** academia, clinical practice, coping strategies, newly appointed nurse educators, transition

## Abstract

**Background:** Transition from clinical to academia requires newly appointed nurse educators to deploy certain coping strategies to thrive in their newly assumed roles. This is because this period is often coupled with high teaching expectations, research, increased workloads, and a lack of proficiency with work–life balance, resulting in a lack of coping. Therefore, this study aimed to explore and describe the coping strategies used by newly appointed nurse educators in a South African university. **Methods:** A qualitative, explorative, and descriptive research design was used. Data were gathered from 12 newly appointed nurse educators using online semi-structured individual interviews. Nurse educators who joined academia from clinical practice within the last six months to five years were included in this study. Data were analysed using Cresswell and Cresswell’s five steps for data analysis. **Findings:** Four categories emerged from the data: newly appointed nurse educators’ experiences of problem-focused coping, emotion-focused coping, meaning-focused coping, and coping through support from others during the transition from clinical practice to academia. Among the many coping strategies, the participants expressed that they apply clinical experience, self-assertiveness, conflict management, and setting boundaries to cope with the transition to academia. **Conclusions:** The findings of this study suggest that the transition from clinical practice to academia remains challenging for newly appointed nurse educators. This study further suggests that there is a need to have support measures in place for newly appointed nurse educators during their transition to academia to improve their ability to cope.

## 1. Introduction

To prepare future nurses for practice, Nursing Education Institutions (NEIs) need experienced clinical nurses to transition to academia [1]. Although teaching is regarded as a fundamental element of good clinical practice, expert nurse clinicians are not automatically competent nurse educators because expertise in bedside nursing does not guarantee proficiency in academia [2]. NEIs need to understand the transitional challenges faced by novice nurse educators during their transition from clinical practice to academia to adequately prepare and retain them [3]. The transition from practice to academia is mostly hectic and challenging, especially when clinical nurses move into academic roles with insufficient training and preparation [4], thus deepening the stress and challenges related to transitioning from clinical settings to academia as they are not knowledgeable about the expectations [5] and consequently leading to a lack of coping with the transition to academia.

Stress is a sign of unmet needs and a leading distraction from the self-actualising process [6]. As a result, individuals with prolonged stress end up having difficulties thriving in different aspects of their lives, thus being unable to reach their full potential [6]. Work-related stress is considered a significant cause of employee illness across all employment settings [7], and newly appointed nurse educators are no exception as they face extremely high levels of work-related stress [8]. What is more alarming is that nurse educators experience higher levels of stress compared to nurses working strictly in the clinical setting [8]. This might be due to high teaching expectations, research, increased workloads, and personal life balance problems that nurse educators encounter at institutions of higher learning [9].

In a study conducted in the United Kingdom by Carr [10], newly appointed nurse educators found the transition into academia stressful, mainly due to the career change from being expert nurse clinicians to nurse educators. This is supported by Barnes and Veesart [11] in their study that explains that transitioning into a nurse educator role can be frustrating, especially if the process is not well explained. The failure to provide clarity in roles during the transition leaves new employees feeling frustrated and contributes massively to additional stress [12]. As a result, newly appointed nurse educators are left with a feeling of being thrown into the role with little training or mentoring [11]. This is also due to the new set of responsibilities and expectations nurse educators are faced with in their new roles [13]. Thus, this period is perceived as challenging and can result in the early departure of newly appointed nurse educators to pursue other job opportunities [14]. Some of the newly appointed nurse educators are more likely to leave academia within five years of being appointed [15]. This usually happens when they do not feel secure in their new positions [16]. This is the rationale that prompted the researcher to conduct the present study.

To thrive in academia, newly appointed nurse educators need to adopt some self-inspired coping strategies to reach their full potential [17]. According to Ab Latif and Nor [18], coping strategies refer to people’s behavioural and psychological efforts to conquer, reduce, or tolerate stressful events. These stabilising methods assist individuals in maintaining psychological adaption during stressful events [19]. In the context of this study, coping can be best understood as newly appointed nurse educators being able to navigate the adversities that accompany the transition to academia and thus be retained longer than 5 years in their newly found roles. According to the literature, some of the coping strategies used by newly appointed nurse educators include orientation and mentoring, problem-focused coping, acceptance, social support, and positive appraisal [20,21,22,23]. However, these coping strategies seem short-lived because the attrition rate within five years of employment of new academic staff members remains alarming to date. Hence, this study sought to explore coping strategies used by newly appointed educators at a South African university.

## 2. Materials and Methods

This study followed a qualitative, explorative, and descriptive design to explore and describe the coping strategies used by newly appointed nurse educators during the transition from clinical practice to academia [24,25,26]. Based on the nature of this study, which was mainly to explore the participants’ experiences with the transition to academia and how they cope with it, this study was grounded in a phenomenological framework. This design assisted the researcher in gaining an in-depth detailed explanation of the study phenomenon, exploring and describing the phenomenon from the participants’ perspective.

### 2.1. Study Setting

This study was conducted in a tri-campus South African university. However, this study was conducted on two campuses where newly appointed nurse educators are based. Although the two campuses are aligned and fall within the same directorship in one school, they are different in terms of geographic locations. One campus is in a predominantly rural area, whereas the other is in a semi-urban area. However, the differences in urbanisation of the campuses had no implication on how newly appointed nurse educators transition to academia.

### 2.2. Participants

The accessible population was nurse educators with a minimum qualification master’s degree in nursing and an additional qualification in nursing education; newly employed nurse educators with at least 6 months to 5 years and employed as lecturers at a South African university were the target population. Participation was limited to an employment experience of at least six months to allow sufficient time for interaction with the transition support offered by the university. Furthermore, a time limit of 5 years was utilized because the literature indicates a higher likelihood of resignation within the first five years of joining academia, as mentioned above [15].

A total population sampling technique was used in this study due to the small size of the newly appointed nurse educators in both campuses where this study was conducted. According to Rai and Thapa [27], total population sampling is a type of purposive sampling technique where the entire population with a particular set of characteristics, such as specific experience, knowledge, and skills, is chosen for the study. This sampling technique is used when the size of the population of interest to the researcher is small [27]. Recruitment and selection of participants and negotiation of paper-based informed consent were conducted by an independent research assistant to avoid undue influence since the researcher is employed at the same institution where the data was collected.

The sample size of this study was determined by the richness of gathered data, which was ensured by collecting data until saturation was reached. However, data saturation was only to be considered when at least six or more participants had been interviewed in each campus [28]. The inclusion criteria were set as follows: (i) newly appointed nurse educator in a South African university and (ii) a minimum of six months to a maximum of five years of being appointed as a nurse educator. The exclusion criteria of this study were set as follows: (i) senior lecturers, associate professors, full professors, and (ii) administration support. The rationale for excluding these categories of academics was that they have acquired more than five years’ experience in the academic field, which could have led to their promotional positions, even though they might be new at the selected university where this study was conducted. The rationale for excluding administration staff was that they were not knowledgeable about the phenomenon of this study. Participation was entirely autonomous, without any influence of cultural, institutional, or local policy factors.

### 2.3. Data Collection

Data were collected through individual online interviews using a semi-structured format. Participants chose the physical space and suitable time to connect to MS teams to ensure that inconvenience was minimised. Data was collected between July and August 2024 by an independent research assistant to avoid bias as the researcher is an employee at the selected university. The independent research assistant is knowledgeable about qualitative data collection based on the track record for qualitative data collection. In addition, the independent research assistant had a master’s degree in nursing and was a PhD candidate at the time of conducting this study. Furthermore, the research assistant underwent ethics training to conduct research with human participants. The online platform for data collection was chosen due to its flexibility when there are space and time constraints, as the two campuses are geographically apart. The independent research assistant requested participants’ consent to use the video camera and record the proceedings of the meeting to be able to ensure the confirmability of the findings. This increased the trustworthiness of this study.

Participants were asked questions relating to coping strategies used by newly appointed nurse educators in a South African university (see Supplementary Materials). This was performed following the interview schedule developed by the researcher. No identifying information, such as participants’ names, identity numbers, or staff numbers, was asked during the interviews to ensure the anonymity of the findings. Rather, participants were referred to as participant 1, 2, 3, et cetera [29].

Participants’ responses were recorded using the recording function on Microsoft Teams, and non-verbal cues were observed through a video feed. Clarity was sought where necessary, thus allowing the participants to elaborate more. Probing and follow-up questions were asked to search for more expanded and detailed information on the topic from the participants. A summary of the participant’s responses was created by the independent research assistant to guarantee to the participant that the independent research assistant was paying attention. Interviews lasted approximately 30 to 60 min, and no follow-up interviews were conducted.

During the interviews, participants were requested to keep their video cameras on to enable the independent research assistant to take notes, as they contain a narrative account of what is happening, that is, the observed events and conversations [29,30]. The independent research assistant wrote notes immediately after an observation was made. Arrangements for interviews were made with participants who voluntarily agreed to participate in this study for data collection based on their availability. Numbers were allocated to participants after the interview to ensure that the report with the participants was not lost and their identities were protected. Data was collected at a convenient time determined by the participants, which was either during lunch breaks or after normal working hours. Participants were requested to be in a quiet place, with signage indicating that the interview was in progress displayed on a closed door to minimise interruptions.

### 2.4. Trustworthiness

Trustworthiness was ensured through credibility, dependability, confirmability, transferability, and authenticity [31]. Measures of triangulation were used during data collection through interviews, observations of participants during interviews, and collection of field notes from the video feed to confirm the findings. The researcher ensured the audit trail was achieved by keeping a comprehensive trace of all research processes. Furthermore, the research methodology was explained in detail in case there are other researchers interested in conducting similar studies and following the same methodology. Data analysis was performed by the researcher and independent co-coder, referred to as investigator triangulation, thus ensuring confirmability of the findings. The researcher ensured that thick description was achieved by thoroughly describing the research context, participants’ experiences, and data collection process and writing a final report. Coping strategies of newly appointed nurse educators from the two campuses were thoroughly described through the data collection process and writing of a final report. The independent research assistant created rapport with participants through prolonged engagement, thus contributing to authenticity.

### 2.5. Data Analysis

Data were analysed following inductive content analysis. The interviews were transcribed verbatim by the researcher before data analysis. Both the second and third authors were available on standby should there be conflict regarding agreement on the categories between the researcher and co-coder. However, no conflicts were encountered between the researcher and the co-coder. Following data transcription, the researcher and a skilled independent co-coder analysed the data independently following the five steps of data analysis by Creswell and Creswell [32] as presented in Table 1.

## 3. Results

The findings of this study are presented according to demographic data as well as categories and sub-categories.

### 3.1. Demographic Data

Data were collected from twelve participants (seven from campus A and five from campus B). The demographic characteristics of the participants are indicated in Table 2.

Demographic data shows that most of the participants were females (nine), with three male participants. The dominance of female participants was attributable to the faculty demographics where this study was conducted, reflecting the fact that nursing has always been a profession that is mostly dominated by females [33]. The age of participants ranged between 25 and 51 years, with the mean age of 39.1 years.

### 3.2. Categories and Sub-Categories

Four categories and sub-categories were identified from the data analysis and are presented in Table 3.

#### 3.2.1. Category 1: Newly Appointed Nurse Educators’ Experiences of Problem-Focused Coping

The first category, newly appointed nurse educators’ experiences of problem-focused coping, had eight sub-categories, namely adaptation through the application of clinical experience; adaptation through understanding and learning; anticipation and being proactive; self-assertiveness, conflict management, and setting boundaries; consultation with others; self-empowerment; time management; and reliance on previous experiences.

The sub-categories are described in this section.

##### Sub-Category 1.1: Adaptation Through the Application of Clinical Experience

Participants expressed that the application of clinical experience played a major role in their transition from clinical practice to academia. They explained that the knowledge acquired during the years spent in clinical practice before moving to academia assisted them in their new environment. They further explained that, even though their transition was not smooth, the experience acquired while in clinical practice helped, as they integrated what they learned at the facilities to navigate their way through. Participants noted that, even though things are conducted differently in academia, their acquired experience from the clinical facilities helped them through their transition. The following are verbatim quotes illustrating this:

*“So then moving from the clinical practice into lecturing, there wasn’t any much of a difference because it was like application of what I have been doing in practice into the theoretical learning. Even when I am teaching in class, the examples that I give are what I experienced during the time I was in the clinical setting, so being in the clinical setting and doing lecturing go hand in hand.”*

Participant 2_Campus A (Female)

*“Hence, I am saying I think it is important to have clinical experience before coming to academia. You know even when you are in clinical you meet the students. All along you are teaching them and you are explaining things to them.”*

Participant 2_Campus A (Female)

##### Sub-Category 1.2: Adaptation Through Understanding and Learning

Participants indicated that they coped by adapting through understanding and learning the way things are conducted in academia. Their explanation of adapting through understanding and learning was centred around understanding the university environment and understanding students’ behaviour and their different learning styles, thus making it easier for them to adapt. They further indicated that understanding how things work reduces the pressure that comes with the transition from clinical practice to academia:

*“Okay, the first thing was to adapt to the new working environment. Adapt to the culture of the university, adapt to how things are done in the university. That was the first thing that I used to cope like going through the university’s academic rules to understand how this institution runs things. Yes, I will say that. So yes, adaptation.”*

Participant 1_Campus A (Female)

*“So, I have learned that I need to understand how they think, how they behave, what is causing their behaviour, and I need to also like be aligned with how they are. You understand?”*

Participant 2_Campus B (Female)

##### Sub-Category 1.3: Anticipation and Being Proactive

Participants stated that anticipation and being proactive also play a major role during the transition from clinical practice to academia. They explained that being proactive when you are in academia makes life easier because you do not know what to expect and you do not need to wait until the last minute to do things. You must be prepared for whatever the students might throw at you. They further explained that this helps them to keep up with the latest trends and be in a better position to impart their knowledge to students.

*“So, whenever I was going to class, I would practice so that I could be perfect before going to stand in front of the students.”*

Participant 1_Campus A (Female)

*“Already you ask yourself questions to say okay if the person does not understand 123, This will be the answer. This is how I will elaborate further for them to understand better. That is part of the planning. You plan by studying.”*

Participant 2_Campus A (Female)

##### Sub-Category 1.4: Self-Assertiveness, Conflict Management, and Setting Boundaries

Participants expressed that the transition from clinical practice to academia required self-assertiveness, conflict management, and setting boundaries. They describe that it is important to put self first and to know conflict management as the period of transition that encompasses dealing with staff and students from different backgrounds as well as knowing when to say no. They explained that this helped them lessen the burden that comes with the transition from clinical practice to academia:

*“I was that person who would always prioritise others before me. Like I will be busy with something and then you come in and you ask me to do something. I would stop whatever I am doing to accommodate you. So, I had to set the boundaries to say I need to have the limit to say no.”*

Participant 1_Campus A (Female)

*“Oh, before the last question. I wanted to add that even the conflict resolution also helped me because I used to have a colleague, and we know we are from different backgrounds. One morning she will be like, I do not want to talk to you. So, the conflict resolution experience that I have acquired helped me to cope because I did not want to work with somebody who was angry or who did not want to communicate with me.”*

Participant 1_Campus A (Female)

##### Sub-Category 1.5: Self-Empowerment

Participants expressed that self-empowerment played a major role during their transition from clinical practice to academia. They further explained that reading the university academic rules and attending webinars and workshops equipped them with the relevant knowledge to deal with issues in the working environment accordingly:

*“I went through the University Academic Rules by myself one by one to see what was expected of me and what was expected of them. For example, a student is constantly missing hours, without reporting, this is what I can do, you know.”*

Participant 2_Campus B (Female)

*“So, what made me able to cope was attending workshops that assisted me in understanding the dynamics of my new work environment.”*

Participant 5_Campus B (Female)

##### Sub-Category 1.6: Time Management

Time management was identified as one of the coping strategies by the participants. Participants expressed that good time management played a major role in coping with transitioning from clinical practice to academia. They expressed that, even though there is a lot of work that needs to be performed, with good time management, one can navigate through their busy schedule and meet their deadlines:

*“The other coping strategy that I used was having a schedule on how to do your things, because if you just work without a schedule, you might end up missing something or you might end up missing a deadline as well.”*

Participant 1_Campus A (Campus A)

*“When I talk about organising yourself, for instance, if I know that I have a class tomorrow, I shouldn’t book myself to be part of the marketing team because I know meetings are on Tuesdays, and my classes are also on Tuesdays.”*

Participant 3_Campus A (Male)

#### 3.2.2. Category 2: Newly Appointed Nurse Educators’ Experiences of Emotion-Focused Coping

The second category was newly appointed nurse educators’ experience of emotion-focused coping, which denotes a process whereby an individual tries to reduce the stressors that they cannot cope with. This category, newly appointed nurse educators’ experiences of emotion-focused coping, had three sub-categories, namely internal drive, self-motivation, and passion; acceptance; and self-confidence.

##### Sub-Category 2.1: Internal Drive, Self-Motivation, and Passion

Participants indicated that internal drive, self-motivation, and passion served as their coping strategies during their transition from clinical practice to academia. They further stated that, even though things were hard, their desire to succeed, their never give up mentality, and their passion kept them going and assisted them with coping during their transition:

*“But then I had to tell myself that, I had a reason why I wanted to move from the clinical to the nursing educational institution. So, I need to overcome my fears and adapt to that environment.”*

Participant 1_Campus A (Female)

*“So yes, it was mixed emotions and a bit scary amongst other things. I wanted to quit within my first three months, but then I said no, I’m not a quitter. Let me just hold on, yeah.”*

Participant 7_Campus A (Female)

##### Sub-Category 2.2: Acceptance

Participants described that acceptance of their positions played a major role in their transition despite the challenges that they faced. They explained that, although they were not involved in decision-making concerning their duties, accepting others’ decisions served as a coping strategy for them. They further explained that acceptance comes with maturity, which helped them to conduct themselves professionally even though they were not happy with not being involved in decision-making:

*“Without consulting me, so I will just leave it like that. She has agreed already meaning there is nothing more for me to add to that. It will not be nice when I say if something happens, go back to the lecture because you agreed with the lecturer in the first place, and I was excluded when you were making those decisions.”*

Participant 1_Campus B (Female)

*“The most important thing is to respect each other and don’t make another person feel inferior. That’s the bottom line. For me to cope, I have allowed the situation to be as it is and it is working for me. Hence, I am still here.”*

Participant 7_Campus A (Female)

##### Sub-Category 2.3: Self-Confidence

Participants expressed that self-confidence played a major role during their transition from their clinical practice to academia. They stated that they used to have low self-confidence while at the clinical practice, but going to an institution of higher learning lifted their self-confidence significantly, making it possible for them to survive in their new working environment:

*“I used to be a shy person so going to the institution of higher learning made me overcome that personality of being shy. I have gained confidence.”*

Participant 1_Campus A (Female)

*“The nice thing is that as much as I am doing administrative work now, when I’m going to hospitals, I have that skill and I’m confident enough to work with patients.”*

Participant 3_Campus B (Male)

#### 3.2.3. Category 3: Newly Appointed Nurse Educators’ Experiences of Meaning-Focused Coping

The third category, newly appointed nurse educators’ experiences of meaning-focused coping, had two sub-categories as described below.

##### Sub-Category 3.1: Appreciation and Being Positive

Another coping strategy used by participants was appreciation and being positive. They stated that, despite the challenges they encountered, knowing that they make a difference in the students’ lives and their work environment is conducive, which was sufficient for them. They further indicated that receiving positive feedback from students kept them going and assisted them to cope with their transition from clinical practice to academia. They also expressed that, as much as they are overwhelmed by students’ behaviour, recalling that they were once students made it easier for them to deal with students’ behaviour. Furthermore, participants stated that, even if they are not appreciated for the work they do, if the working environment is conducive, that is sufficient:

*“I said to myself I need to make a difference in their lives. As much as I feel overwhelmed by their behaviour then it means I’m the one who needs to change their behaviour.”*

Participant 2_Campus B (Female)

*“Those are small waters under the bridge, so you don’t have to worry about those things you are getting paid. You come to work every day, you do your work and there is no one bullying you when you come to work. So those are the things that we should be grateful for.”*

Participant 7_Campus A (Female)

##### Sub-Category 3.2: Religious Coping

Participants expressed that another coping strategy that assisted them with their transition was prayer. They expressed that, whenever they felt like things were getting hectic and there was no one to turn to because everyone was swamped with their work, prayer was their way to cope. That is where they gathered their strength in difficult times:

*“It was a lot for me but thankfully I am a prayerful person. I am a Christian, so I was praying most of the time for the Almighty to give me the strength to overcome those challenges. I was depending on God more for guidance throughout this process. Whenever I felt overwhelmed, I would kneel, pray, and read my word and after that, I would feel encouraged and revived.”*

Participant 1_Campus B

*“Yes, you need to be connected. It keeps you rooted mmm, because prayer is about communication with the sources that are above you. As I said the workload is a lot, so you need something that is above you, above that workload that you link up with. Yeah, to give you that strength to replenish your energies that are failing, perhaps, or that are threatened because of the load.”*

Participant 6_Campus A

#### 3.2.4. Category 4: Newly Appointed Nurse Educators’ Coping Through Support from Others

The fourth category, newly appointed nurse educators’ coping through support from others, had five sub-categories, namely orientation and induction, organisational and managerial support and capacitation, mentoring and role modelling, support from colleagues and teamwork, and support from family.

##### Sub-Category 4.1: Orientation and Induction

Participants expressed that orientation and induction helped them with their transition from clinical practice to academia. They expressed that the transition was a bit challenging for them at first because of the new environment, but orientation and induction made it easier for them to settle into their new environment. They stated that, as much as there were challenges during their transition, there were also a lot of positives, which included orientation and induction, to take them through this challenging journey. They also explained that, even though there is orientation and induction, they wish that the timeframe could be extended so that it can give them ample time to adjust to their new environment:

*“So, transitioning for me was a bit challenging at first of which I think with the new orientation that is being implemented at my institution now, it made things better for me because I could follow a simple guide that this is what I’m supposed to do. That is where I had to adjust a lot because, you know, sitting behind the desk sometimes can be exhausting.”*

Participant 3_Campus B (Male)

*“As much as there were challenges, there were also a lot of positives as well because you have your orientation period, you have your induction then you get to know how things are working in the learning institution.”*

Participant 5_Campus B

##### Sub-Category 4.2: Organisational and Managerial Support and Capacitation

Participants noted that organisational and managerial support and capacitation played a significant role in their transition. They further expressed that, even though you might be having challenges with having to adjust in the new environment, if the resources are made available for you and you have support from the manager, this lessens the pressure that comes with transition:

*“Yes, so being welcomed alone gives one a positive perspective on the new career or the new field they have chosen. Therefore, if there is support, I believe one will have to also do introspection to say do I want to stay in academia or not. Let me just give it a try for this year or a few years if I’m still not enjoying it then I can go back to clinical practice.”*

Participant 3_Campus B (Male)

*“The management also encourages you to attend more workshops that are related to your work meaning, the workshops that focus on the new guidelines that are coming forth and clinical programmes workshops. You are also encouraged to attend workshops related to research because when you are in the university you also must do research.”*

Participant 5_Campus A (female)

##### Sub-Category 4.3: Mentoring and Role Modelling

Participants expressed that mentoring and role modelling were strategies that assisted them to cope with the transition to academia. However, they indicated that, even though mentoring and role modelling were not formalised to some of them, they played a major role in their transition. Participants indicated that consulting with other faculty members who came before them greatly aided them to transition to academia. They further described that, although they had challenges with technology, especially since not everyone is exposed to computers at the clinical facilities, having a person whom they sought clarity from helped significantly. They mentioned that having someone knowledgeable in the working environment to take them through their journey played a major role:

*“I also had a mentor through this process. I used to work closely with her, and then she would mentor me on how we do things. She was very helpful yes. For me to call her mentor is because of the way she presented herself towards me so that is why I call her a mentor. I’m not even sure that she was aware that I took her as a mentor.”*

Participant 1_Campus A (Female)

*“The fact that there were other lecturers who kind of mentored me, although it was indirect mentoring, I could ask them a lot of questions”*

Participant 4_Campus B (Female)

##### Sub-Category 4.4: Support from Colleagues and Teamwork

Participants stated that the support from colleagues and teamwork helped them with their transition from clinical practice to academia. They expressed that the words of encouragement and patience from other colleagues made their transitioning experience less challenging. They further stated that, when things were not going accordingly, sharing with their team members always helped them take off the load:

*“Luckily what assisted me cope was that I was working with someone. The person also just came in I think a month before me, so we were able to teach each other the things that we are supposed to do or how to cope with work when we are stressed. The colleague that I was working with, I think that’s the one that assisted me to cope with how to do the job and all of that.”*

Participant 4_Campus A (Female)

*“The culture of being professional, kind, and empathetic is also a good trait that I think all professionals need to have, irrespective of power. You know at the clinical, people will tell you no address me by a doctor or I am the matron. Don’t call me by my surname or just Miss or whatever. But now when you get to academia, they will tell you no my name is Faith. I’m Hope, or I’m whoever. Anything that has to do with the title don’t worry about it.”*

Participant 3_Campus B (Male)

##### Sub-Category 4.5: Support from Family

Participants stated that the support from family also helped them with their transition from clinical practice to academia. They expressed that having the support of those close to you while trying to adjust to your new working environment is a morale booster. They further expressed that, when things were not going accordingly, sharing with their family members and being assured that we are with you every step helped them lessen the pressure:

*“They don’t make noise in the house and at least they have prepared lunch for you. It’s nice when you get there and they just don’t bother you and if you need assistance or anything, they are there to support you. Are you fine? Okay, I made you some tea. You just relax when you come back, you talk, you laugh.”*

Participant 3_Campus B (Male)

*“So frustrating. I even called my niece to say how are you coping with these electronic things, technology, and everything? And she was saying, you are so smart. You are going to do fine, just hang in there everything will fall into place, but it was tough for me.”*

Participant 7_Campus A (Female)

## 4. Discussion

This study aimed at exploring and describing the coping strategies used by newly appointed nurse educators during the transition from clinical practice to academia at a South African university. Data were collected from the newly appointed nurse educators with five years or less of experience. The major findings of this study were that newly appointed nurse educators experience problem-focused coping, emotion-focused coping, meaning-focused coping, and coping through support from others. The findings of this study are congruent with Lazarus and Folkman’s 1984 Transactional Model of Stress and Coping, which highlights that, although stress is primarily a result of a person’s interaction with their environment, problem-focused and emotion-focused coping strategies are key to navigate through the stressors [34].

### 4.1. Experiences of Problem-Focused Coping

Participants expressed that their experiences of problem-focused coping meant taking control of the situation and making it work despite the challenges. This is supported by the findings of a study conducted by Carrol [35] that describes problem-focused coping as coping that seeks to resolve the stressful situation or change the source of stress, which was utilised by the participants in this study. Participants also expressed that they had to learn to adapt to theoretical and clinical demands and balance the two during their transition. They highlighted that not only were they required to strike a balance between the two, but they also had social demands to consider, thus making their transition more challenging. They also described that their experiences of problem-focused coping were also accompanied by proper time management and reliance on previous experience. A study conducted by Muazzam et al. [36] reported similar findings; they described that problem-focused coping strategies are effective as people facing challenges in their workplace are enabled and empowered to confront those challenges. In this case, the challenge was the difficulty in adapting to the new academic environment. Based on the findings of this study, the authors agree that, even though the transition from clinical practice to academia is challenging, problem-focused coping might serve as an ideal coping strategy for newly appointed nurse educators during this period.

### 4.2. Experiences of Emotion-Focused Coping

A study conducted by Aulén et al. [37] describes emotion-focused coping as the feelings that occur because of the problem. Consistent with this study, participants explained their experiences of emotion-focused coping as making efforts to reduce stressors that they cannot cope with during their transition. They expressed that, even though their main goal was to balance theoretical and clinical demands, they also needed to be involved in other activities, such as community engagement and research, at the institution of higher learning. They further expressed that not only did this take a toll on them emotionally, but it created more stress since there was no feedback given to determine whether they were on the right track with their work or not. Participants indicated that it was through internal drive, passion, self-motivation, acceptance, and self-confidence that they managed to cope with their transition. Based on the findings of this study, it can be said that emotion-focused coping can serve as an effective coping for newly appointed nurse educators during their transition from clinical practice to academia.

### 4.3. Experiences of Meaning-Focused Coping

Guo et al. [38] described that meaning-focused coping does not attempt to change a tough situation nor does it directly reduce the burden caused by the distress. Rather, it aims to change the evaluation of the situation and make beliefs, goals, and stressful situations more consistent so that individuals are more open to dealing with stressful situations [38]. Similar findings are seen in this study, where participants explained their experiences of meaning-focused coping as remaining positive and hoping for better days despite being overwhelmed by their challenges. Participants explained that staying focused while knowing the difference they make in the lives of the students was priceless. They stated that changing how they perceived the situation they found themselves in made them look forward to the next day despite the challenges. They also mentioned that prayer played a major role during their transition because that is where they gathered their strength whenever they were feeling overwhelmed and could not cope. Considering the findings of this study, the authors agree that, even though the transition from clinical practice to academia is challenging, meaning-focused coping might serve as an effective coping strategy for newly appointed nurse educators.

### 4.4. Coping Through Support from Others

The participants showed the need for effective coping strategies due to the challenges they faced during the transition from clinical practice to academia. A study conducted by Salimzadeh et al. [39] shows that academic staff have been found to experience high levels of work-related stress, which is no exception for newly appointed nurse educators. Participants stated that, even though they managed to stay in academia despite the challenges they faced, their wish is for the duration of orientation and induction as well as mentoring and role modelling to be reviewed. This is supported by the study conducted by the same authors, Salimzadeh et al. [39], that found engagement coping, which includes, amongst other things, seeking support, to be an active strategy to deal directly with stressful situations. This resonates with the findings of this study, where participants indicated that they had to seek support from other colleagues to cope. Furthermore, participants stated that, even though they attended orientation and induction at the university, it was more of a general orientation and did not help much with what was expected from them in their new roles. They mentioned that it was not sufficient, as they found themselves having to seek clarity time and again from their colleagues. They felt like they were an unnecessary burden to other colleagues, which resulted in them fearing to seek assistance, especially because there was no specific person allocated to them. This is supported by the findings of a study conducted by Raub et al. [40], which states that effective employee orientation is important in helping new employees understand both specific and general requirements concerning their job role. Participants also expressed that not being allocated a mentor formally made their transition challenging, as they had to rely on more than one person for guidance. This is supported by similar findings of a study conducted by Shanks et al. [41], which described mentoring as one of the most important components of the induction programme. This is a process whereby newly appointed educators are guided by a more experienced employee to acquire knowledge about teaching practices and develop their own professional identity [41]. This was not the case with the participants of this study, as they expressed that they were not properly mentored. The findings of this study call for a departmental-specific orientation programme as well as the allocation of a mentor for newly appointed nurse educators to help with a smooth transition from clinical practice to academia.

## 5. Conclusions

The transition of newly appointed nurse educators from clinical practice to academia remains challenging, and most employees do not cope well during this period. Thus, the transition from clinical practice to academia needs effective coping strategies. The findings of this study highlighted newly appointed nurse educators’ experiences of problem-focused coping, emotion-focused coping, meaning-focused coping, and coping through support from others. The findings show that there is a need to provide all the necessary support for newly appointed nurse educators during their transition from clinical practice to academia, especially in the first five years after joining academia. Such support can be achieved through re-designing context-specific orientation programs, structured mentorship, as well as the promotion of a culture of job support backed by academic directors and human resourced directorate.

## Figures and Tables

**Table 1:** Adapted Creswell and Creswell steps for data analysis.

Data Analysis Steps	Description of Application in this Study
Step 1: Organise and prepare the data for analysis	The researcher transcribed recorded data verbatim for analysis.
Step 2: Read or look at all the data	The researcher and co-coder read all data from all the interviews to have a general sense of information and reflect on the overall meaning of the data.
Step 3: Code all the data	Gathered data was segmented into sentences and labelling categories to bring about data codes. This process was performed by the researcher and co-coder independently using Atlas.ti version 24.
Step 4: Generate a description and categories	The coding process was used to generate a description of the setting as well as the categories and sub-categories, which are reported later in this paper as major findings.
Step 5: Represent the description and categories	The researcher narrated the results using a detailed discussion of several categories and sub-categories (see Results Section).

**Table 2:** Demographic characteristics of participants.

Participant Demographics	Campus A	Campus B
Total number of participants	7	5
**Gender**		
Male	2	1
Female	5	4
**Age (years)**		
25–30	1	2
31–35	2	
36–40	1	
41–45	1	1
46–50	1	2
51–55	1	-
**Years of experience in academia**		
0–1	1	2
1–2	-	2
2–3	-	
3–4	3	1
4–5	3	

**Table 3:** Categories and sub-categories.

Categories	Sub-Categories
1. Newly appointed nurse educators’ experiences of problem-focused coping	1.1. Adaptation through the application of clinical experience 1.2. Adaptation through understanding and learning 1.3. Anticipation and being proactive 1.4. Self-assertiveness, conflict management, and setting boundaries 1.5. Self-empowerment 1.6. Time management
2. Newly appointed nurse educators’ experiences of emotion-focused coping	2.1. Internal drive, self-motivation, and passion 2.2. Acceptance 2.3. Self-confidence
3. Newly appointed nurse educators’ experiences of meaning-focused coping	3.1. Appreciation and being positive 3.2. Religious coping
4. Newly appointed nurse educators’ coping through support from others	4.1. Orientation and induction 4.2. Organisational and managerial support and capacitation 4.3. Mentoring and role modelling 4.4. Support from colleagues and teamwork 4.5. Support from family

## Data Availability

The datasets used and analysed during the current study are available from the corresponding author on reasonable request. Data are not publicly available due to privacy and ethical restrictions.

## Supplementary Materials

The following supporting information can be downloaded at: https://www.mdpi.com/article/10.3390/nursrep15100367/s1, Data collection tool/Interview schedule.

## Public Involvement Statement

There was no public involvement in any aspect of this research.

## Guidelines and Standards Statement

This manuscript was drafted against the SRQR (Standards for Reporting Qualitative Research [42].

## Abbreviations

The following abbreviations are used in this manuscript:

NEI	Nursing education institution
PhD	Doctor of philosophy
