# Supplementary material for: Coping Strategies Used by Newly Appointed Nurse Educators During Transition from Clinical Practice to Academia: A Qualitative Study
Source: Nurs Rep. 2025 Oct 15;15(10):367. doi: 10.3390/nursrep15100367 (PMC12567427; doi:10.3390/nursrep15100367)
Supplement: Supplementary file 1 [file nursrep-15-00367-s001.zip › nursrep-3889150-supplementary.pdf]

**Supplementary Materials: Data collection tool/Interview schedule**

NWU®
NORTH-WEST UNIVERSITY
NOORDWES-UNIVERSITEIT
UNIBESITI YA BOKONE-BOPHIRIMA

Coping strategies used by newly appointed nurse educators in a South African university

Type of interview : Virtual individual interview using either Google Meet, Microsoft Teams, Zoom or WhatsApp

Interview venue : Virtually

Participants number: Pseudo-names

<u>Question to be asked</u>

- Please tell me your experience as a newly appointed nurse educator in this institution.
- How do you cope as a newly appointed nurse educator in this institution?
- What can be done to assist you to assist you cope?
- Is there anything that you would like to add?

- Follow up questions will be determined by the participants' responses.
- Probing follow up questions will be used to search for more comprehensive information from participants to elaborate more on the topic.
- Summary of the responses from the participants will be done to reassure the participants that the researcher is paying attention.
